# Antimicrobial susceptibility and genetic characteristics of multi-drug resistant *Acinetobacter baumannii* isolates in Northwest China

**DOI:** 10.3389/fmicb.2024.1293725

**Published:** 2024-04-30

**Authors:** Meimei Hu, Hongjia Sun, Yanmei Xu, Xiaoying Xu

**Affiliations:** Medical Laboratory Center, The First Hospital of Lanzhou University, Lanzhou, China

**Keywords:** *Acinetobacter baumannii*, carbapenem resistance, CC208, MDR, molecular epidemiology, ST469

## Abstract

**Introduction:**

In recent decades, widespread multi-drug resistant (MDR) bacteria have become a serious problem in healthcare facilities.

**Methods:**

To systematically summarize and investigate the prevalence and genomic features of clinical MDR *Acinetobacter baumannii* (*A. baumannii*) clinical isolates recovered from the first hospital of Lanzhou University, we collected 50 MDR *A. baumannii* isolates isolated in the first quarter of 2022 and using whole-genome sequencing investigate the genotypic characteristics.

**Results:**

All of these isolates were generally resistant to the common β-lactamase antibiotics. Resistance to cefoperazone-sulbactam varies greatly between different clones. The proportion of CC208 isolates resistant and mediated to cefoperazone-sulbactam is as high as 84.6%. There were no isolates resistant to tigecycline and colistin. The presence of *bla*_OXA − 23_ (94.0%) and *bla*_OXA − 66_ (98.0%) were the most frequent determinants for carbapenem resistance. Two main endemic clones were identified, one (ST469^oxf^) was predominantly circulating in ICUs and carried the same resistance genes, virulence genes and transposons, and the other clone (CC208) carried more resistance genes and had more widely disseminated.

**Discussion:**

Our study showed that clinical MDR *A. baumannii* isolates circulating in our hospital exhibited highly similar genetic features. We should take timely and effective measures to control the further epidemic of these isolates.

## Introduction

*Acinetobacter baumannii* (*A. baumannii*) is an important Gram-negative pathogen that often causes serious nosocomial infections, especially among immunocompromised and elderly patients in intensive care units (ICUs) (Sarshar et al., 2021). It has been estimated that between 47% and 93% of *A. baumannii* infections are associated with multi-drug resistance (MDR), which is facilitated through a variety of well-documented mechanisms (β-lactamases, efflux pumps, aminoglycoside-modifying enzymes, permeability defects, and target modifications) (Gallagher and Baker, 2020). In China, the epidemic of *A. baumannii* in the ICUs is even more severe. Research showed that 71.4% of ICUs were found to be contaminated by carbapenem-resistant *A. baumannii* strains (Liu et al., 2022). A study reported a 10-fold rise in the incidence of *A. baumannii*–related bloodstream infections in China during 2009–2018, from 99 to 926 per 1,000,000 ICU populations (Meng et al., 2021). Another research showed that *A. baumannii* was the most frequent bacterial isolate in ventilator-associated pneumonia in China, and rates were 35.7%−52.7% (Xie et al., 2018).

Carbapenem antibiotics have historically been considered a first-line treatment for *A. baumannii* infections (Poirel et al., 2011). Data from the Antimicrobial Surveillance Network (CHINET) showed that the proportion of *A. baumannii* isolates that are resistant to meropenem and imipenem has reached nearly 72% in China during 2022. Several β-lactamases can cause carbapenem resistance in *A. baumannii*, but the most common are class D β-lactamases, including those belonging to the OXA-23,−24,−51, and−58 families (Poirel and Nordmann, 2006; Walther-Rasmussen and Hoiby, 2006).

Multidrug resistance, especially carbapenem resistance in *A. baumannii*, is a global healthcare concern. There was a lot of research on MDR *A. baumannii* in China. However, there was little available data on the phenotypic and genotypic characteristics of MDR *A. baumannii* isolates in northwest China. To investigate the prevalence and genomic features of MDR *A. baumannii* transmitted in the first hospital of Lanzhou University, which is a large tertiary hospital, a total of 50 MDR *A. baumannii* isolates were collected and analyzed by whole-genome sequencing (WGS).

## Materials and methods

### Bacterial isolates and clinical data

We collected all *A. baumannii* isolates isolated from people who were hospitalized in the first quarter of 2022 in the first hospital of Lanzhou University, Gansu Province, China. Specimens from the same site in the same patient were removed. We further screened for MDR *A. baumannii* from these isolates. MDR *A. baumannii* was defined by resistance to three or more representatives of the following classes of antibiotics: quinolones, extended-spectrum cephalosporins, β-lactam/β-lactamase inhibitor combination, aminoglycoside, and carbapenems (Hujer et al., 2006). In the end, we collected 31 ICU-derived isolates and 19 other department-derived isolates in chronological order. If the patient has a history of ICU exposure within 1 month, we also classify the isolates as ICU-derived. The following clinical data were collected: age, gender, the main indicator of inflammation within 48 h of MDR *A. baumannii* positive (Zhang et al., 2021), primary disease, hospitalization time duration, outcome at discharge, and administration of antibiotics.

### Susceptibility testing

All *A. baumannii* isolates were isolated from people who were hospitalized in the first hospital of Lanzhou University. *A. baumannii* isolates were identified by matrix-assisted laser desorption-ionization-time of flight mass spectrometry (MALDI-TOF MS; BioMérieux, Marcy l'Etoile, France). Minimal inhibitory concentrations (MICs) of Piperacillin-tazobactam, Cefoperazone-sulbactam, Ceftazidime, Cefepime, Imipenem, Meropenem, Ciprofloxacin, Levofloxacin, Trimethoprim-sulfamethoxazole, Amikacin, Tobramycin, Tigecycline, Minocycline and Colistin were tested by the VITEK-2 compact automatic microbial analyzer (BioMérieux, Marcy l'Etoile, France). The reference strains, *Pseudomonas aeruginosa* ATCC 27853 and *Escherichia coli* ATCC 25922, are being used as quality control. Ampicillin-sulbactam (10/10 μg) was tested for susceptibility using a disk diffusion test. Interpretations of resistance phenotypes follow those of the Clinical and Laboratory Standards Institute (CLSI) guideline document M100-S32. There is no susceptibility breakpoint for *Acinetobacter* to cefoperazone-sulbactam has been provided by CLSI, we referred to CLSI breakpoints for *Enterobacteriaceae* susceptibility and resistance to cefoperazone-sulbactam. The criteria for the susceptibility of tigecycline were adapted from the U. S. Food and Drug Administration.

We calculated the multiple antibiotics resistance (MAR) index for all samples. MAR index was calculated as described by Blasco et al. (2008) as follows: MAR = *a*/*b*, where *a* = number of antibiotics to which the isolate was resistant; *b* = total number of antibiotics against which individual isolate was tested.

### Whole genome sequencing and bioinformatics analysis

The total bacterial DNA of 50 clinical MDR *A. baumannii* isolates was extracted using TIANamp bacteria DNA kit (TIANGEN, Beijing, China), following the manufacturer's instructions. For testing sample qualification, the DNA concentration was measured by NanoDrop 2000 (Thermo Fisher Scientific, The United States of America). The extracted DNA was sent to Zhejiang Tianke Biotechnology Co., Ltd., for whole-gene sequencing. MLST of the test isolates based on the public databases for molecular typing and microbial genome diversity (https://pubmlst.org/) using the Oxford scheme (Jolley et al., 2018). The genetic relatedness of STs was analyzed and visualized using PHYLOVIZ 2.0a goeBURST analysis (Nascimento et al., 2017). For sequence analysis and annotation, the BLAST algorithm (http://www.ncbi.nlm.nih.gov/BLAST) and the RAST server (https://rast.nmpdr.org/) were utilized (Overbeek et al., 2014). The antimicrobial resistance (AMR) genes were identified using the comprehensive antibiotic resistance database (CARD; https://card.mcmaster.ca/) (Alcock et al., 2020). The transposon regions and the integrons were investigated using BacAnt (http://www.bacant.net/BacAnt/) (Hua et al., 2021). Also, the virulence factor genes (VFGs) were detected using the virulence factor database (VFDB; http://www.mgc.ac.cn/cgi-bin/VFs/v5/main.cgi) (Chen et al., 2005).

### Phylogenetic analysis

One of the isolates we isolated LZU2 (ST469^oxf^) was used as the reference strain. Single nucleotide polymorphisms (SNPs) alignment was matched using package Snippy v4.6.0, and phylogenetic trees were constructed by fasttree v2.1.10. Cytoscape v3.7.2 software was used to visualize the phylogenetic tree.

### Statistical analysis

Quantitative data of normal distribution were presented as mean deviation, while those of abnormal distribution were expressed as the median and quartile. Qualitative data were presented as a percentage (%) and their inter-group comparison was performed with a chi-square test, qualitative data incapable of being analyzed with a chi-square test were compared between two groups using Fisher's exact test. SPSS 27.0 software was used for statistical analysis, and *P* < 0.05 indicated a statistically significant difference.

## Results

### Patient demographics and clinical profiles

In this study, we collected a total of 50 isolates, including 21 isolates from the ICU, 10 isolates from the emergency intensive care unit (EICU), and 19 isolates from other wards. The cardiac surgery ward had the highest separation rate among wards excluding intensive care units (16.0%).

The clinical profiles showed that, of the 50 patients infected with MDR *A. baumannii*, 80.0% were male, pulmonary disease was the most common primary disease, these patients had a median age of 59 years, the median total length of hospital stay was 33 days and 44% of cases died. These isolates were collected mostly from sputum and accounted for 75% of all samples (Table 1).

**Table 1:** General information statistics.

	**Total (*n* = 50)**
Age (years), *M*, (IQR)	59 (48,72)
**Gender (%)**	
Male	40 (80.0)
Female	10 (20.0)
**Specimen source (%)**	
Sputum	39 (78.0)
Abdominal fluid	1 (2.0)
Broncho-alveolar lavage	2 (4.0)
Blood	3 (6.0)
Catheter	1 (2.0)
Pus	1 (2.0)
Secretion	2 (4.0)
Tracheal aspirate	1 (2.0)
**The main indicator of inflammation within 48 h**	
WBC (10^9^/L) (*M*)	12.25
Neutrophils (%) (*M*)	82.61
PCT (ng/ml), *M*, (IQR)	0.642 (0.162, 2.290)
**Classification of primary disease (%)**	
Cerebrovascular disease	4 (8.0)
Heart diseases	6 (12.0)
Pulmonary disease	13 (26.0)
Intracranial infection	2 (4.0)
Trauma	6 (12.0)
Aortic dissection	5 (10.0)
Tumors	3 (6.0)
Multiple organ failure	3 (6.0)
Others	8 (16.0)
Hospitalization time duration (days), *M*, (IQR)	33 (16.5, 63)
**Outcome at discharge (%)**	
Survive	28 (56.0)
Dead	22 (44.0)

We investigated antibiotic use strategies 1 week before and after MDR *A. baumannii* was detected in these patients. The survey showed that the top three antibiotic strategies were carbapenem alone, cefoperazone-sulbactam alone, and piperacillin-tazobactam alone.

### Susceptibility testing

Antibiotic susceptibility testing was performed on the 50 MDR *A. baumannii* isolates. Just as revealed by drug resistance test results, these isolates were generally resistant to the common β-lactamase antibiotics, the rate of resistance to ampicillin-sulbactam, piperacillin-tazobactam, ceftazidime, and cefepime were 100%, 98.0%, 96.0%, and 86.0%, and 92.0% isolates were found resistance to both imipenem and meropenem. Resistance to Cefoperazone-sulbactam is relatively low, at 20%. Most of the isolates were quinolone resistant, 98.0% of isolates were resistant to ciprofloxacin, and 92.0% of isolates were resistant to levofloxacin. The rate of resistance to trimethoprim-sulfamethoxazole, amikacin and tobramycin were 30%, 44%, and 68%, respectively. Additionally, only 6.0% of isolates were resistant to minocycline, but 28.0% of isolates showed intermediate resistance to minocycline. There were no isolates resistant to tigecycline and colistin. The details are shown in Table 2. We calculated the MAR index for all samples. The MAR index was higher than the 0.2 limit in all our tested isolates (Table 3).

**Table 2:** The results of drug resistance test and genetic characteristics.

**Categories of antibiotics**	**Antimicrobial agent**	**Total (*****n*** = **50)**		
		***S*** **(%)**	***I*** **(%)**	***R*** **(%)**
β-lactams	Ampicillin-sulbactam	0 (0)	0 (0)	50 (100)
	Piperacillin-tazobactam	1 (2.0)	0 (0)	49 (98.0)
	Cefoperazone-sulbactam	26 (52.0)	14 (28.0)	10 (20.0)
	Ceftazidime	1 (2.0)	1 (2.0)	48 (96.0)
	Cefepime	1 (2.0)	6 (12.0)	43 (86.0)
	Imipenem	4 (8.0)	0 (0)	46 (92.0)
	Meropenem	3 (6.0)	1 (2.0)	46 (92.0)
Quinolone	Ciprofloxacin	1 (2.0)	0 (0)	49 (98.0)
	Levofloxacin	2 (4.0)	2 (4.0)	46 (92.0)
Folate inhibitors	Trimethoprim-sulfamethoxazole	33 (66.0)	2 (4.0)	15 (30.0)
Aminoglycosides	Amikacin	28 (56.0)	0 (0)	22 (44.0)
	Tobramycin	16 (32.0)	0 (0)	34 (68.0)
Tetracycline	Tigecycline	45 (90.0)	5 (10.0)	0 (0)
	Minocycline	33 (66.0)	14 (28.0)	3 (6.0)
Polymyxins	Colistin	50 (100)	0 (0)	0 (0)

**Table 3:** Multiple antibiotics resistance (MAR) index of the MDR *Acinetobacter baumannii* isolates.

**MAR index**	**No. (%)**
0.3	1 (2.0)
0.5	14 (28)
0.6	14 (28)
0.7	18 (36)
0.8	3 (6)

### MLST analysis

In our study, the *gdhB* allele in the ST_Oxford scheme had two copies in most isolates except ST931. A total of eight STs were detected for the 50 MDR *A. baumannii* isolates by MLST, including ST469, ST208/1806, ST195/1816, ST540, ST368/1962, ST369/1837, ST931 and ST2336. ST469 was predominant in all MDR *A. baumannii* isolates (46.0%), followed by ST208/1806 (18.0%), ST195/1816 (14.0%), ST540 (14.0%), ST368/1962 (2.0%), ST369/1837 (2.0%), ST931 (2.0%) and ST2336 (2.0%).

**Figure 1:**
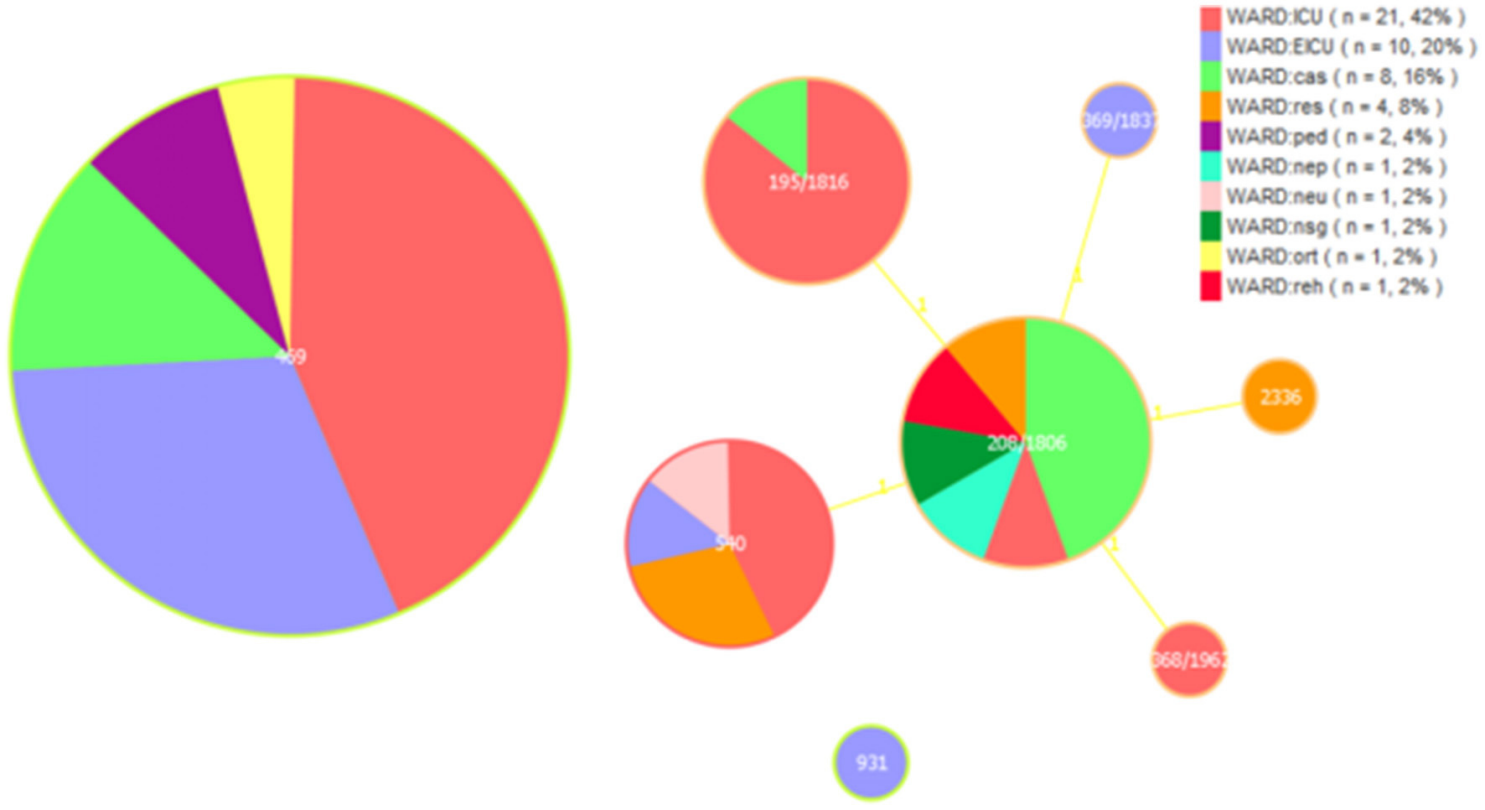
Multi-locus sequence typing (MLST) and minimum spanning tree (MST) population analysis of 50 MDR *Acinetobacter baumannii* isolates from the different clinical departments. Minimum spanning tree (MST) based on STs profiles of 50 MDR *A. baumannii* isolates. Each circle represents an ST and the size of the circle is proportional to the number of isolates. Different colors represent different wards. ICU, intensive care unit; EICU, emergency intensive care unit; ped, pediatric ward; ort, orthopedics ward; res, respiratory medicine ward; reh, rehabilitation ward; neu, neurology ward; nsg, neurosurgery ward; nep, nephrology ward; cas, cardiac surgery ward.

We analyzed ST types distributed in different departments. We found that ST469 is mainly distributed in the ICU and EICU, and ST195/1816 are almost exclusively present in the ICU. ST208/1806 is mainly found in departments outside the ICU and EICU. Among these isolated clones, ST208/1806, ST195/1816, ST540, ST368/1962, ST369/1837, and ST2336 were SLVs and all these STs belonged to clonal complex 208 (CC208; Figure 1).

### Genetic characteristics and phylogenetic analysis

WGS analysis demonstrated that most MDR *A. baumannii* isolates harbored carbapenemase genes *bla*_OXA − 23_ (94.0%) and *bla*_OXA − 66_ (98.0%). Cephalosporinase genes *bla*_ADC30_ (36.0%) and *bla*_ADC73_ (62.0%) are also expressed in most isolates. These isolates tend to carry one of the two genes. The 44% of isolates carried *bla*_TEM − 1_, which encodes resistance to β-lactams. A total of 8 genes [*AAC(3)*-Ia, *AAC(6')*-Ib9, *APH(6)*-Id, *ANT(3”)*-IIc, *APH(3')*-Ia, *APH(3”)*-Ib, *aadA, armA*] encoding aminoglycoside resistance were found in these isolates. The 98.0% of isolates carried *APH(6)*-Id, *ANT(3”)*-IIc and *APH(3”)*-Ib. All of these isolates contained the *tet* (B) and *tet*R genes predicting tetracycline resistance and *gyrA*^S81L^, *parC*^S84L^, *parC*^V104I^ and *parC*^D105E^ genes encoding fluoroquinolone resistance. The *sul1* and *sul2* genes that encode resistance to sulfonamide were observed in 30.0% and 10.0% isolates, respectively. In addition, 84.0% of isolates possessed the *mphE* and *msrE* genes encoding macrolide resistance. We also found other genes such as the *LpsB* (100%) and *catB8* (20.0%) genes, which provide resistance to peptide antibiotics and chloramphenicol, respectively. In addition to these genes, the resistance-nodulation-cell division (RND) antibiotic efflux pump and major facilitator superfamily (MFS) antibiotic efflux pump also played an important role in antibiotic resistance.

Overall, the most isolates carried Tn6292, Tn2007, Tn6022-delta-1, Tn6205, Tn6166, the presence is 84%, 94%, 88%, 98%, and 84%, respectively. Twenty-eight percent of isolates possessed Tn2009 and mainly being detected in CC208 isolates. We detected a total of eight integrons in these isolates.

We further analyzed the existence of virulence factor genes. All of the MDR *A. baumannii* isolates harbored genes *ade*FGH, *bap, csu*ABCDE and *pga*ABCD (associated with biofilm formation), *plc* and *plcD* (encoding phospholipase), *lpsB and lpxABCDLM* (associated with immune evasion), *barAB, basABCDFGHIJ, bauABCDEF, entE and hemO* (related to iron uptake), *abaI and abaR* (played a role in Quorom sensing), *bfmR and bfmS* (controlled biofilm formation and cellular morphology), *pbpG* encoding penicillin-binding protein, *OmpA* associated with adherence. The 96% of isolates possessed virulence factor *katA* associated with stress adaption. The distribution of *pilE* varies from isolate to isolate. Only 46% of isolates expressed the *pilE* gene which was essential for twitching motility and natural competence and contributed to host cell adherence.

**Figure 2:**
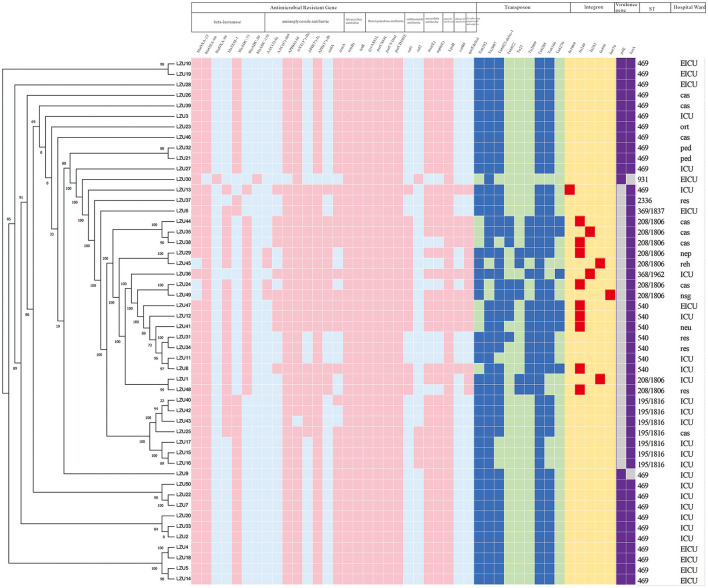
Phylogenetic analysis and heat map of the detection of ARGs, transposons, integrons and VFGs among the 50 MDR *Acinetobacter baumannii* clinical isolates. The left side is phylogenetic tree constructed by calling SNPs from core gene alignment of the study 50 MDR *A. baumannii* clinical isolates. Pink, blue, red, purple colors represent express the corresponding gene, respectively. Light blue, light green, yellow, gray colors represent absent of the corresponding gene, respectively. ICU, Intensive care unit; EICU, emergency intensive care unit; ped, pediatric ward; ort, orthopedics ward; res, respiratory medicine ward; reh, rehabilitation ward; neu, neurology ward; nsg, neurosurgery ward; nep, nephrology ward; cas, cardiac surgery ward. Because all of these isolates harbored the following virulence factor genes *adeFGH, bap, csuABCDE, pgaABCD, plc, plcD, lpsB, lpxABCDLM, pbpG, bfmRS, abaI, abaR, barAB, basABCDFGHIJ, bauABCDEF, entE, OmpA*, we only shown two genes, *pilE* and *katA*.

We mapped the heat map of resistance genes, transposons, integrons and virulence factor genes (*pilE* and *katA*; Figure 2). Combined with phylogenetic analysis and molecular characteristics, we found that the MDR *A. baumannii* prevalent in our hospital consists mainly of two clonal populations: ST469, ST208 and its SLVs (CC208). We found that ST469 and CC208 have large differences in genomic characteristics. CC208 isolates carried more resistance genes. Except LZU13, all ST469 isolates possessed the same resistance genes and transposons.

### Comparative analysis between the ST469 group and CC208 group

We compared the differences in resistance gene carriers between the ST469 group (*n* = 23) and the CC208 group (*n* = 26; Table 4). We found that the carrying rates of the following genes in CC208 group: *bla*_TEM − 1_, *bla*_ADC30_, *AAC(6')-Ib9, APH(3')-Ia, aadA, sul1, catB8* and *qacEdelta1* were significantly higher than those in ST469 group, while the carrying rates of *bla*_ADC73_ and *armA* in ST469 group were significantly higher than those in CC208 group. Furthermore, the ST469 isolates harbored the same transposon elements, Tn6292, Tn2007, Tn6022-delta-1, Tn6025, and Tn6166. Most CC208 isolates harbored both Tn2007 and Tn2009 elements. We further analyzed the differences in antibiotic susceptibility phenotypes between the two groups (Table 5), and showed that the nonsensitivity rates of cefoperazone-sulbactam and trimethoprim-sulfamethoxazole were significantly higher in the CC208 group than those in the ST469 group. The proportion of CC208 isolates resistant and mediated to cefoperazone-sulbactam was as high as 84.6%.

**Table 4:** Comparative analysis of the antimicrobial resistance genes in ST469 group and CC208 group.

**Categories of antibiotics**	**Antimicrobial resistance genes**	**ST469 (*n* = 23)**	**CC208 (*n* = 26)**	***P***
Beta-lactamase	*bla*__*O*_XA − 23_	23 (100)	23 (88.5)	0.237
	*bla*_OXA − 66_	23 (100)	26 (100)	–
	*bla*_OXA − 98_	0	0	–
	*bla*_TEM − 1_	1 (4.3)	21 (80.8)	< 0.001
	*bla*_ADC73_	22 (95.7)	9 (34.6)	< 0.001
	*bla*_ADC30_	1 (4.3)	17 (65.4)	< 0.001
	*bla*_ADC158_	0	0	–
Aminoglycoside antibiotic	*AAC(3)-Ia*	0	4 (15.4)	0.112
	*AAC(6')-Ib9*	1 (4.3)	9 (34.6)	0.009
	*APH(6)-Id*	23 (100)	26 (100)	–
	*ANT(3”)-IIc*	23 (100)	25 (96.2)	1.000
	*APH(3')-Ia*	1 (4.3)	20 (76.9)	< 0.001
	*APH(3”)-Ib*	23 (100)	26 (100)	–
	*aadA*	1 (4.3)	14 (53.8)	< 0.001
	*armA*	23 (100)	16 (61.5)	< 0.001
Tetracycline antibiotic	*tet (B)*	23 (100)	26 (100)	–
	*tetR*	23 (100)	26 (100)	–
Fluoroquinolone antibiotic	*gyrA^*S*81*L*^*	23 (100)	26 (100)	–
	*parC^*S*84*L*^*	23 (100)	26 (100)	–
	*parC^*V*104*I*^*	23 (100)	26 (100)	–
	*parC^*D*105*E*^*	23 (100)	26 (100)	–
Sulfonamide antibiotic	*sul1*	1 (4.3)	14 (53.8)	< 0.001
	*sul2*	0	4 (15.4)	0.112
Macrolide antibiotic	*msr(E)*	23 (100)	19 (73.1)	0.011
	*mph(E)*	23 (100)	19 (73.1)	0.011
Peptide antibiotic	*LpsB*	23 (100)	26 (100)	–
Phenicol antibiotic	*catB8*	1 (4.3)	9 (34.6)	0.009
Disinfecting agents and antiseptics	*qacEdelta1*	1 (4.3)	14 (53.8)	< 0.001

**Table 5:** Comparative analysis of antibiotic nonsensitivity rates in ST469 group and CC208 group.

**Antimicrobial agent**	**ST469 (*n* = 23)**	**CC208 (*n* = 26)**	***P***
Ampicillin-sulbactam	23 (100)	26 (100)	–
Piperacillin-tazobactam	23 (100)	25 (96.2)	1.000
Cefoperazone–sulbactam	2 (8.7)	22 (84.6)	< 0.001
Cefepime	23 (100)	25 (96.2)	1.000
Ceftazidime	23 (100)	25 (96.2)	1.000
Imipenem	23 (100)	22 (84.6)	0.112
Meropenem	23 (100)	23 (88.5)	0.237
Levofloxacin	22 (95.7)	25 (96.2)	1.000
Ciprofloxacin	23 (100)	25 (96.2)	1.000
Amikacin	7 (30.4)	15 (57.7)	0.056
Minocycline	8 (34.8)	9 (34.6)	0.990
Trimethoprim-sulfamethoxazole	2 (8.7)	14 (53.8)	< 0.001
Tobramycin	16 (69.6)	18 (69.2)	0.980
Colistin	0 (0)	0 (0)	–
Tigecycline	1 (4.3)	4 (15.4)	0.353

## Characterization of carbapenemase-encoding genes

**Figure 3:**
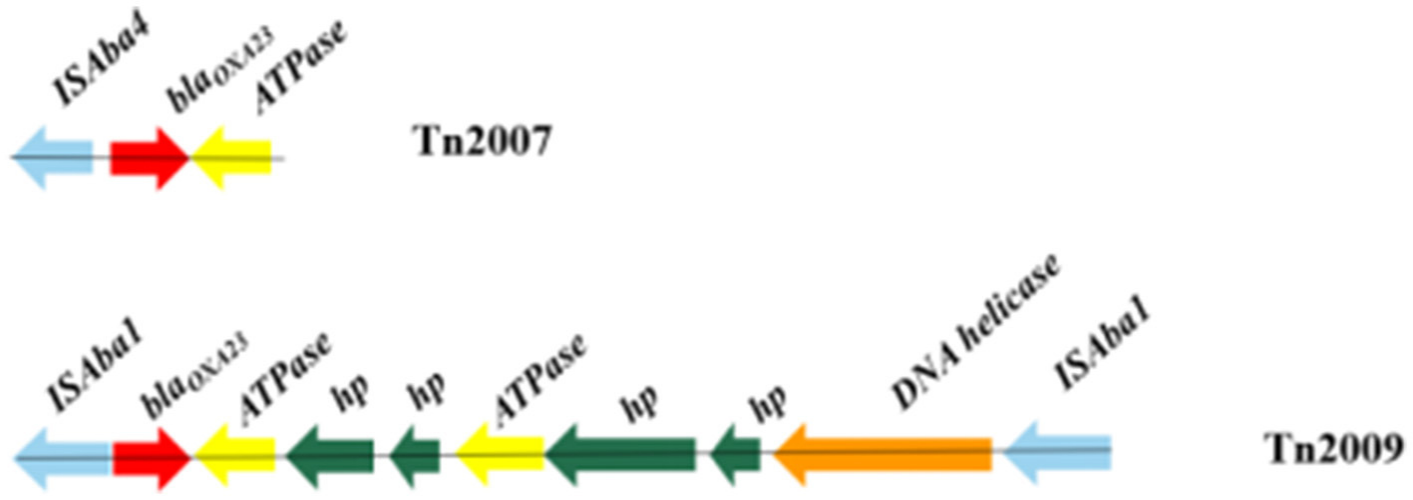
Genotypic characterization of *bla*_OXA − 23_ in *Acinetobacter baumannii*. Carbapenemase genes are represented by red arrows, and other coding sequences are represented with other different colors.

A variety of carbapenemase encoding genes were found in these MDR *A. baumannii* isolates, the presence of *bla*_OXA − 23_ (94.0%) and *bla*_OXA − 66_ (98.0%) was the most frequent determinants for carbapenem resistance. BLASTN indicated that the *bla*_OXA − 23_ gene resided exclusively in the Tn2007 and Tn2009 elements. Tn2007 harbors the *bla*_OXA − 23_ gene and a single copy of *ISAba4* upstream of the *bla*_OXA − 23_. The structure of Tn2009 was *ISAba1*-*bla*_OXA − 23_-*ATPase-hp-hp-ATPase-hp-hp-DNA helicase-ISAba1*, which has two copies of *ISAba1* flanking an internal segment containing *bla*_OXA − 23_ (Figure 3).

## Discussion

A comparatively small number of pathogens are responsible for a substantial burden of hospital-acquired infections globally, *A. baumannii is one* of the key pathogens (Gallagher and Baker, 2020). Carbapenem antibiotics have historically been considered a first-line treatment for *A. baumannii* infections. However, according to the literature, increased resistance to carbapenems in the *A. baumannii* strain has been observed globally over the past decade (Rodríguez et al., 2018; Teerawattanapong et al., 2018). Carbapenem resistance in *A. baumannii* is medicated by several coexisting mechanisms, while the most prevalent mechanism is associated with the production of oxacillinases (Ambler class D β-lactamases), especially OXA-23 (Li et al., 2019; Nordmann and Poirel, 2019). This work also confirms that the production of the OXA-type carbapenem-hydrolyzing-class D β-lactamase is the key mechanism for carbapenem resistance in *A. baumannii*. In our study, the detection of resistant genes showed that the most common carbapenemase-resistant gene was *bla*_OXA − 66_ (98.0%) in all MDR *A. baumannii* isolates, followed by *bla*_OXA − 23_ (94.0%). Research showed that without the promoter provided by *ISAba1*, the OXA-23 carbapenemase does not confer clinically relevant levels of resistance to carbapenem antibiotics (Walther-Rasmussen and Hoiby, 2006; Figueiredo et al., 2009). *ISAba1* has been shown to provide a strong promoter that drives a high level of expression of *bla*_OXA − 23_ (Segal et al., 2007). To date, five transposons containing the *bla*_OXA − 23_ gene, including Tn2006, Tn2007, Tn2008, Tn2008B, and Tn2009, have been reported (Nigro and Hall, 2016). In our study, Tn2007 was identified in 94.0% of isolates. However, only 28.0% of isolates possessed Tn2009 and only being detected in CC208 isolates. BLASTN indicated that Tn2009 has two copies of *ISAba1* flanking an internal segment containing *bla*_OXA − 23_. There is currently no evidence that Tn2007 is a transposon that is capable of self-movement (Nigro and Hall, 2016). Therefore, Tn2009 may played a major role in the horizontal transfer of *bla*_OXA − 23_ in the CC208 isolates isolated from our hospital. Gao et al. (2022) described the potential circular intermediate of Tn2009 and suggested that replicative transposition contributes to the evolution and transmission of OXA-23-producing ST208. This structure Tn2009 element has only been reported in China (Zhou et al., 2011; Zhu et al., 2013). Previous research showed that Tn2009 and Tn2008 played dominant roles in the dissemination of *bla*_OXA − 23_ in China (Wang et al., 2011; Liu et al., 2015). In this study, Tn2006 and Tn2008 were not detected in these isolates.

There is still no optimal therapeutic regimen for MDR *A. baumannii* infection, sulbactam-based regimens, tigecycline-based regimens, and polymyxins-based regimens have been recommended as the main treatment for MDR *A. baumannii* infection in China (Group et al., 2016). Fortunately, resistance to tigecycline and polymyxin has not been found in MDR *A. baumannii* isolated from our hospital, so combination therapy based on tigecycline and polymyxin is recommended. As for the use of cefoperazone-sulbactam, our study showed that there was a large difference in resistance rates between different clones. The proportion of CC208 isolates resistant and mediated to cefoperazone-sulbactam is as high as 84.6% in our study. Therefore, the use of cefoperazone-sulbactam as empiric treatment regimens in patients with CC208 MDR *A. baumannii* infection is not recommended. Recently, a research about bloodstream infection caused by MDR *A. baumannii* showed that the most effective antibacterial regimen was the combination of cefoperazone-sulbactam and tigecycline (Yu et al., 2021). It was worth noting that the MDR *A. baumannii* isolated in our hospital has a very low resistance rate to minocycline. As the treatment options of MDR *A. baumannii* infections are considerably limited, reinstituting the use of older antimicrobials has now become a priority. Beganovic et al. (2021) evaluate minocycline alone and in combination with other commonly utilized anti-microbials against CRAB and non-CRAB isolates. They found that triple therapy with high-dose minocycline, continuous-infusion sulbactam, and polymyxin B produced the most significant kill against the CRAB, with no regrowth and minimal resistance development. A growing number of studies have shown that minocycline could constitute an alternative agent for the treatment of MDR *A. baumannii* infections (Lashinsky et al., 2017; Fragkou et al., 2019).

In our study, ST469 is the predominant sequence types in ICUs (17/31, 54.8%), followed by ST195/1816 (6/31, 19.4%). This differs from the prevalence of MDR *A. baumannii* in ICUs in other hospitals in China. It has been reported that ST208 and ST195 were the predominant epidemic types of MDR *A. baumannii* in China (Qu et al., 2016; Jiang et al., 2019). However, ST208 is the predominant circulating strain in out-of-ICUs (8/19, 42.1%) in our study. We found that the popular MDR *A. baumannii* isolates in our hospital is divided into two main endemic clones, ST469 and CC208. These two endemic clones have large differences in genomic characteristics. CC208 isolates carried more resistance genes. The carrying rates of the following genes in CC208 group: *bla*_TEM − 1_, *bla*_ADC30_, *AAC(6')-Ib9, APH(3')-Ia, aadA, sul1, catB8* and *qacEdelta1* were significantly higher than those in ST469 group. We further analyzed the differences in antibiotic susceptibility phenotypes between these two groups, and showed that the nonsensitivity rates of cefoperazone-sulbactam were significantly higher in the CC208 group than in the ST469 group. Previous studies have shown that the *bla*_TEM − 1_ and *bla*_ADC30_ could confer sulbactam resistance to *A. baumannii* (Krizova et al., 2013; Kuo et al., 2015). The current research on the mechanism of sulbactam resistance is very limited, our study provided evidence that *bla*_TEM − 1_ and *bla*_ADC30_ is associated with *A. baumannii*. resistance to sulbactam. We also found that CC208 isolates had a significantly higher resistance rate to trimethoprim-sulfamethoxazole than ST469 isolates, possibly because most CC208 isolates carry the *sul1* gene. Moreover, previous study shown that CC208 isolates was more tolerant to various adverse environments than other isolates (Bian et al., 2021). CC208 isolates has strong resistance to antibiotic and the environment at the same time, so it is very important to rapid identify the CC208 isolates and take some infection control measures to reduce the spread of CC208 MDR *A. baumannii*.

Recently, discussions arose about the accuracy of the two *A. baumannii* multilocus sequence typing (MLST) schemes (Castillo-Ramirez and Grana-Miraglia, 2019). Stefano Gaiarsa et al. reported issues of the Oxford scheme regarding *gdhB* paralogy, recombination, primer sequences, and position of the genes on the genome (Gaiarsa et al., 2019). The same problem was encountered in our study, the *gdhB* allele in the ST_Oxford scheme had two copies in most isolates except LZU30. However, according to our research, MLST still has its unique advantages in bacterial homologous analysis. The Oxford scheme shows higher concordance with phylogenies. But we think that this approach should be improved. As on multiple occasions, allele sequences resulting from this duplication were incorrectly used to establish new Oxford STs that do not exist, Stefano Gaiarsa et al. suggest that these *gdhB*-based alleles should be removed from the database (Castillo-Ramirez and Grana-Miraglia, 2019). We maintain the same opinion. The wrong calling of alleles at *gdhB* locus would artifactually inflated the diversity recorded using the Oxford scheme.

In conclusion, we present a whole-genome-based investigation of the prevalence and genomic features of MDR *A. baumannii* clinical isolates in a large tertiary hospital in Gansu. The most common carbapenemase-resistant genes still was *bla*_OXA − 66_ and *bla*_OXA − 23_ in our hospital. Two main endemic clones were identified in our hospital. One clone was predominantly circulating in ICUs and exhibited the same genetic characteristics (ST469), and another clone (CC208) carried more resistance genes and had more widely disseminate. The spread of CC208 isolates may pose a greater challenge to the treatment of MDR *A. baumannii* infection. We should take timely and effective measures to control the further epidemic of these isolates.

## Data availability statement

The data supporting this work are available under NCBI bioproject PRJNA1103124.

## Ethics statement

The studies involving humans were approved by the Ethics Committee of LZU No.1 hospital. The studies were conducted in accordance with the local legislation and institutional requirements. The participants provided their written informed consent to participate in this study.

## Author contributions

MH: Conceptualization, Data curation, Formal analysis, Investigation, Methodology, Writing – original draft, Writing – review & editing. HS: Funding acquisition, Supervision, Writing – review & editing. YX: Investigation, Methodology, Writing – review & editing. XX: Funding acquisition, Supervision, Writing – review & editing.

## References

[B1] Alcock B. P. Raphenya A. R. Lau T. T. Tsang K. K. Bouchard M. Edalatmand A.. (2020). CARD 2020: antibiotic resistome surveillance with the comprehensive antibiotic resistance database. Nucleic Acids Res. 48, D517–D525. 10.1093/nar/gkz935 31665441 PMC7145624

[B2] Beganovic M. DaffineeK E. Luther M. K. LaPlante K. L. (2021). Minocycline alone and in combination with polymyxin B, meropenem, and sulbactam against carbapenem-susceptible and -resistant *Acinetobacter baumannii* in an *in vitro* pharmacodynamic model. Antimicrob. Agents Chemother. 65, e01680-20. 10.1128/AAC.01680-20 33318006 PMC8092495

[B3] Bian X. Liu X. Zhang X. Li X. Zhang J. Zheng H.. (2021). Epidemiological and genomic characteristics of *Acinetobacter baumannii* from different infection sites using comparative genomics. BMC Genomics 22:649. 10.1186/s12864-021-07942-2 34247587 PMC8272988

[B4] Blasco M. D. Esteve C. Alcaide E. (2008). Multiresistant waterborne pathogens isolated from water reservoirs and cooling systems. J. Appl. Microbiol. 105, 469–475. 10.1111/j.1365-2672.2008.03765.x 18298535

[B5] Castillo-Ramirez S. Grana-Miraglia L. (2019). Inaccurate multilocus sequence typing of *Acinetobacter baumannii*. Emerg. Infect. Dis. 25, 186–187. 10.3201/eid2501.180374 30561303 PMC6302598

[B6] Chen L. Yang J. Yu J. Yao Z. Sun L. Shen Y.. (2005). VFDB: a reference database for bacterial virulence factors. Nucleic Acids Res. 33(suppl_1), D325–D328. 10.1093/nar/gki008 15608208 PMC539962

[B7] Figueiredo S. Poirel L. Croize J. Recule C. Nordmann P. (2009). In vivo selection of reduced susceptibility to carbapenems in *Acinetobacter baumannii* related to ISAba1-mediated overexpression of the natural bla(OXA-66) oxacillinase gene. Antimicrob. Agents Chemother. 53, 2657–2659. 10.1128/AAC.01663-08 19307373 PMC2687192

[B8] Fragkou P. C. Poulakou G. Blizou A. Blizou M. Rapti V. Karageorgopoulos D. E.. (2019). The role of minocycline in the treatment of nosocomial infections caused by multidrug, extensively drug and pandrug resistant *Acinetobacter baumannii*: a systematic review of clinical evidence. Microorganisms 7:159. 10.3390/microorganisms7060159 31159398 PMC6617316

[B9] Gaiarsa S. Batisti Biffignandi G. Esposito E. P. Castelli M. Jolley K. A. Brisse S.. (2019). Comparative analysis of the two *Acinetobacter baumannii* multilocus sequence typing (MLST) schemes. Front. Microbiol. 10:930. 10.3389/fmicb.2019.00930 31130931 PMC6510311

[B10] Gallagher P. Baker S. (2020). Developing new therapeutic approaches for treating infections caused by multi-drug resistant *Acinetobacter baumannii*. J. Infect. 81, 857–861. 10.1016/j.jinf.2020.10.016 33115656

[B11] Gao Y. Li H. Chen H. Zhang J. Wang R. Wang Z.. (2022). Origin, phylogeny, and transmission of the epidemic clone ST208 of carbapenem-resistant *Acinetobacter baumannii* on a global scale. Microbiol. Spectr. 10:e0260421. 10.1128/spectrum.02604-21 35638783 PMC9241911

[B12] Group C. X. C. W. Guan X. He L. Hu B. Hu J. Huang X.. (2016). Laboratory diagnosis, clinical management and infection control of the infections caused by extensively drug-resistant Gram-negative bacilli: a Chinese consensus statement. ClinMicrobiol Infect. 22, S15–S25. 10.1016/j.cmi.2015.11.004 26627340

[B13] Hua X. Liang Q. Deng M. He J. Wang M. Hong W.. (2021). BacAnt: a combination annotation server for bacterial DNA sequences to identify antibiotic resistance genes, integrons, and transposable elements. Front. Microbiol. 12:649969. 10.3389/fmicb.2021.649969 34367079 PMC8343408

[B14] Hujer K. M. Hujer A. M. Hulten E. A. Bajaksouzian S. Adams J. M. Donskey C. J.. (2006). Analysis of antibiotic resistance genes in multidrug-resistant *Acinetobacter* sp. isolates from military and civilian patients treated at the Walter Reed Army Medical Center. Antimicrob. Agents Chemother. 50, 4114–4123. 10.1128/AAC.00778-06 17000742 PMC1694013

[B15] Jiang L. Liang Y. Yao W. Ai J. Wang X. Zhao Z.. (2019). Molecular epidemiology and genetic characterisation of carbapenem-resistant *Acinetobacter baumannii* isolates from Guangdong Province, South China. J. Glob. Antimicrob. Resist. 17, 84–89. 10.1016/j.jgar.2018.11.002 30445207

[B16] Jolley K. A. Bray J. E. Maiden M. C. (2018). Open-access bacterial population genomics: BIGSdb software, the PubMLST.org website and their applications. Wellcome Open Res. 3:124. 10.12688/wellcomeopenres.14826.1 30345391 PMC6192448

[B17] Krizova L. Poirel L. Nordmann P. Nemec A. (2013). TEM-1 beta-lactamase as a source of resistance to sulbactam in clinical strains of *Acinetobacter baumannii*. J. Antimicrob. Chemother. 68, 2786–2791. 10.1093/jac/dkt275 23838947

[B18] Kuo S.-C. Lee Y.-T. Lauderdale T.-L. Y. Huang W.-C. Chuang M.-F. Chen C.-P.. (2015). Contribution of *Acinetobacter*-derived cephalosporinase-30 to sulbactam resistance in *Acinetobacter baumannii*. Front. Microbiol. 6:231. 10.3389/fmicb.2015.00231 26284030 PMC4517069

[B19] Lashinsky J. N. Henig O. Pogue J. M. Kaye K. S. (2017). Minocycline for the treatment of multidrug and extensively drug-resistant *A. baumannii*: a review. Infect. Dis. Ther. 6, 199–211. 10.1007/s40121-017-0153-2 28357705 PMC5446366

[B20] Li S. Duan X. Peng Y. Rui Y. (2019). Molecular characteristics of carbapenem-resistant *Acinetobacter* spp. from clinical infection samples and fecal survey samples in southern China. BMC Infect. Dis. 19:900. 10.1186/s12879-019-4423-3 31660862 PMC6819553

[B21] Liu C. Chen K. Wu Y. Huang L. Fang Y. Lu J.. (2022). Epidemiological and genetic characteristics of clinical carbapenem-resistant *Acinetobacter baumannii* strains collected countrywide from hospital intensive care units (ICUs) in China. Emerg. Microbes Infect. 11, 1730–1741. 10.1080/22221751.2022.2093134 35730377 PMC9258068

[B22] Liu L. L. Ji S. J. Ruan Z. Fu Y. Fu Y. Q. Wang Y. F.. (2015). Dissemination of *bla*OXA-23 in *Acinetobacter spp*. in China: main roles of conjugative plasmid pAZJ221 and transposon Tn2009. Antimicrob. Agents Chemother. 59, 1998–2005. 10.1128/AAC.04574-14 25605357 PMC4356780

[B23] Meng X. Fu J. Zheng Y. Qin W. Yang H. Cao D.. (2021). Ten-year changes in bloodstream infection with *Acinetobacter baumannii* complex in intensive care units in eastern China: a retrospective cohort study. Front. Med. 8:715213. 10.3389/fmed.2021.715213 34422870 PMC8374942

[B24] Nascimento M. Sousa A. Ramirez M. Francisco A. P. Carriço J. A. Vaz C.. (2017). PHYLOViZ 2.0 providing scalable data integration and visualization for multiple phylogenetic inference methods. Bioinformatics 33, 128–129. 10.1093/bioinformatics/btw582 27605102

[B25] Nigro S. J. Hall R. M. (2016). Structure and context of *Acinetobacter* transposons carrying the oxa23 carbapenemase gene. J. Antimicrob. Chemother. 71, 1135–1147. 10.1093/jac/dkv440 26755496

[B26] Nordmann P. Poirel L. (2019). Epidemiology and diagnostics of carbapenem resistance in gram-negative bacteria. Clin. Infect. Dis. 69, S521–S528. 10.1093/cid/ciz824 31724045 PMC6853758

[B27] Overbeek R. Olson R. Pusch G. D. Olsen G. J. Davis J. J. Disz T.. (2014). The SEED and the rapid annotation of microbial genomes using subsystems technology (RAST). Nucleic Acids Res. 42, D206–D214. 10.1093/nar/gkt1226 24293654 PMC3965101

[B28] Poirel L. Bonnin R. A. Nordmann P. (2011). Genetic basis of antibiotic resistance in pathogenic *Acinetobacter* species. IUBMB Life. 63, 1061–1067. 10.1002/iub.532 21990280

[B29] Poirel L. Nordmann P. (2006). Carbapenem resistance in *Acinetobacter baumannii*: mechanisms and epidemiology. Clin. Microbiol. Infect. 12, 826–836. 10.1111/j.1469-0691.2006.01456.x 16882287

[B30] Qu J. Y. Du Y. Yu R. J. Lü X. J. (2016). The first outbreak caused by *Acinetobacter baumannii* ST208 and ST195 in China. Biomed Res. Int. 2016:9254907. 10.1155/2016/9254907 27144176 PMC4842041

[B31] Rodríguez C. H. Nastro M. Famiglietti A. (2018). Carbapenemases in *Acinetobacter baumannii*. review of their dissemination in Latin America. Rev. Argent. Microbiol. 50, 327–333. 10.1016/j.ram.2017.10.006 29548732

[B32] Sarshar M. Behzadi P. Scribano D. Palamara A. T. Ambrosi C. (2021). *Acinetobacter baumannii*: an ancient commensal with weapons of a pathogen. Pathogens 10:387. 10.3390/pathogens10040387 33804894 PMC8063835

[B33] Segal H. Jacobson R. K. Garny S. Bamford C. M. Elisha B. G. (2007). Extended-10 promoter in ISAba-1 upstream of blaOXA-23 from *Acinetobacter baumannii*. Antimicrob. Agents Chemother. 51, 3040–3041. 10.1128/AAC.00594-07 17548500 PMC1932550

[B34] Teerawattanapong N. Panich P. Kulpokin D. Ranong S. N. Kongpakwattana K. Saksinanon A.. (2018). A systematic review of the burden of multidrug-resistant healthcare-associated infections among intensive care unit patients in southeast asia: the rise of multidrug-resistant *Acinetobacter baumannii*. Infect. Control Hosp. Epidemiol. 39, 525–533. 10.1017/ice.2018.58 29580299

[B35] Walther-Rasmussen J. Hoiby N. (2006). OXA-type carbapenemases. J. Antimicrob. Chemother. 57, 373–83. 10.1093/jac/dki482 16446375

[B36] Wang X. Zong Z. Lu X. (2011). Tn2008 is a major vehicle carrying blaOXA-23 in *Acinetobacter baumannii* from China. Diagn. Microbiol. Infect. Dis. 69, 218–222. 10.1016/j.diagmicrobio.2010.10.018 21251570

[B37] Xie J. Yang Y. Huang Y. Kang Y. Xu Y. Ma X.. (2018). The current epidemiological landscape of ventilator-associated pneumonia in the intensive care unit: a multicenter prospective observational study in China. Clin. Infect. Dis. 67(suppl_2), S153–S161. 10.1093/cid/ciy692 30423055

[B38] Yu K. Zeng W. Xu Y. Liao W. Xu W. Zhou T.. (2021). Bloodstream infections caused by ST2 *Acinetobacter baumannii*: risk factors, antibiotic regimens, and virulence over 6 years period in China. Antimicrob. Resist. Infect. Control. 10:16. 10.1186/s13756-020-00876-6 33461617 PMC7814448

[B39] Zhang T. Xu X. Xu C. F. Bilya S. R. Xu W. (2021). Mechanical ventilation-associated pneumonia caused by *Acinetobacter baumannii* in Northeast China region: analysis of genotype and drug resistance of bacteria and patients' clinical features over 7 years. Antimicrob. Resist. Infect. Control. 10:135. 10.1186/s13756-021-01005-7 34526127 PMC8444615

[B40] Zhou H. Zhang T. Yu D. Pi B. Yang Q. Zhou J.. (2011). Genomic analysis of the multidrug-resistant *Acinetobacter baumannii* strain MDR-ZJ06 widely spread in China. Antimicrob. Agents Chemother. 55, 4506–4512. 10.1128/AAC.01134-10 21788470 PMC3187012

[B41] Zhu L. Yan Z. Zhang Z. Zhou Q. Zhou J. Wakeland E. K.. (2013). Complete genome analysis of three *Acinetobacter baumannii* clinical isolates in China for insight into the diversification of drug resistance elements. PLoS ONE 8:e66584. 10.1371/journal.pone.0066584 23826102 PMC3691203

